# Pre-surgical fMRI Localization of the Hand Motor Cortex in Brain Tumors: Comparison Between Finger Tapping Task and a New Visual-Triggered Finger Movement Task

**DOI:** 10.3389/fneur.2021.658025

**Published:** 2021-05-14

**Authors:** Marco Ciavarro, Eleonora Grande, Luigi Pavone, Giuseppina Bevacqua, Michelangelo De Angelis, Paolo di Russo, Roberta Morace, Giorgia Committeri, Giovanni Grillea, Marcello Bartolo, Sergio Paolini, Vincenzo Esposito

**Affiliations:** ^1^Istituto di Ricovero e Cura a Carattere Scientifico (IRCCS) Neuromed, Pozzilli, Italy; ^2^Department of Neuroscience, Imaging and Clinical Sciences, University “Gabriele d'Annunzio” of Chieti-Pescara, Chieti, Italy; ^3^Department of Human Neurosciences, University of Rome “La Sapienza”, Rome, Italy

**Keywords:** hand knob, gliomas, fMRI, pre-surgical planning, motor area

## Abstract

**Introduction:** Pre-surgical mapping is clinically essential in the surgical management of brain tumors to preserve functions. A common technique to localize eloquent areas is functional magnetic resonance imaging (fMRI). In tumors involving the peri-rolandic regions, the finger tapping task (FTT) is typically administered to delineate the functional activation of hand-knob area. However, its selectivity may be limited. Thus, here, a novel cue-induced fMRI task was tested, the visual-triggered finger movement task (VFMT), aimed at eliciting a more accurate functional cortical mapping of the hand region as compared with FTT.

**Method:** Twenty patients with glioma in the peri-rolandic regions underwent pre-operative mapping performing both FTT and VFMT. The fMRI data were analyzed for surgical procedures. When the craniotomy allowed to expose the motor cortex, the correspondence with intraoperative direct electrical stimulation (DES) was evaluated through sensitivity and specificity (mean sites = 11) calculated as percentage of true-positive and true-negative rates, respectively.

**Results:** Both at group level and at single-subject level, differences among the tasks emerged in the functional representation of the hand-knob. Compared with FTT, VFMT showed a well-localized activation within the hand motor area and a less widespread activation in associative regions. Intraoperative DES confirmed the greater specificity (97%) and sensitivity (100%) of the VFMT in determining motor eloquent areas.

**Conclusion:** The study provides a novel, external-triggered fMRI task for pre-surgical motor mapping. Compared with the traditional FTT, the new VFMT may have potential implications in clinical fMRI and surgical management due to its focal identification of the hand-knob region and good correspondence to intraoperative DES.

## Introduction

Preservation of motor function in brain tumor surgery involving the peri-rolandic regions is a challenge for neurosurgeons. Surgical management remains the most successful strategy to date, although the resection of tumors in this region, more than in other eloquent areas, has been historically accompanied by considerable rates of incomplete resection and high risk of morbidity (1). Accurate localization of functional areas around the primary motor cortex (M1) is crucial to reduce negative outcomes while reaching the maximum resection (2). Intraoperative neurophysiological monitoring (IOM) through direct electrical stimulation (DES) is commonly employed. When this region is exposed, DES can be performed with a monopolar stimulation probe; otherwise, the positioning of a subdural strip electrode is required for continuous monitoring of motor evoked potentials (MEPs) during the resection (3). Preoperatively, the anatomical landmark such as the Ω-shaped structure has been traditionally used for the localization of the hand motor area (4). However, mass effects associated with brain tumors can distort these common relations; on the other hand, functional areas may be relocated to other brain regions, making anatomy-based localization of eloquent areas more challenging (5).

In this scenario, a variety of non-invasive pre-operative functional brain mapping techniques are nowadays successful in localizing motor function with a good correspondence with IOM (6). An emerging tool in pre-operative identification of the motor cortex (M1) is the navigated transcranial magnetic stimulation (nTMS) that, similarly to DES, establishes a causal link between the stimulation area and the observed motor outputs (7). Nevertheless, beyond the lack of availability in neurosurgery centers, a major disadvantage is that nTMS may not be employed in those cases in which, due to peritumoral edema and therapies necessary to prevent tumor-related seizures, it is necessary to increase the stimulation intensities to evoke MEPs, exposing the patient at risk for unfathomed events (stimulation-induced seizures and increased stimulation-related discomfort) (8).

By virtue of its non-invasiveness, fMRI is a widely available technique for mapping brain functions. It is considered a powerful tool in the pre-operative planning for surgical procedures involving M1, providing a 92% correspondence to DES mapping data (3, 9, 10) and high percentage of both sensitivity, ranging from 71 to 100%, and specificity, from 68 to 100% (11). It measures brain activity by recording concomitant changes in cerebral perfusion during task execution, and it is considered to be a quicker, less stressful and repeatable method in pre-operative brain mapping. Besides, fMRI allows a more detailed coregistration between structural and functional data (12) that may be useful both in pre-surgical planning, to determine the operative trajectories, and intraoperatively to guide subdural strip electrode positioning, especially in those cases in which the craniotomy does not allow to expose M1. The finger tapping task (FTT) is one of the easier tasks to be performed to investigate functional activation of the hand-knob area. The FTT requires a repetitive self-paced touch of thumb to each finger (13, 14) that is compared with a “no-task” control condition. Although FTT is widely used, the corresponding functional activation map involves not only those regions commonly associated with the execution of voluntary finger movements (i.e., the primary and supplementary motor cortices, basal ganglia, and cerebellum) but also other areas, such as the premotor and somatosensory motor cortices, which may play a more general role in motor tasks (15). In addition, the difficulties in executing the FTT in patients with partial motor deficits may determine poor functional imaging data (16).

In the light of these considerations, we developed a new task for pre-surgical motor mapping, the visual-triggered finger movement task (VFMT), with the aim of overcoming the weakness of FTT. VFMT was indeed designed by requiring simple finger movements without sensory feedback and an active control task during the rest period. We hypothesized that the VFMT, with respect to FTT, may provide a more focal and reliable functional localization of the hand motor region. Thus, it may be able to better predict the spatial relation between the lesion and the eloquent area, being useful in rating the surgical resection entities. We tested our hypothesis in a sample of patients who underwent tumor resection in central areas, by using both motor tasks during pre-surgical mapping.

## Materials and Methods

### Subjects

Of 115 patients with a lesion involving central regions who underwent fMRI investigation of motor functions for pre-operative mapping between December 2017 and August 2020, 20 consecutive patients (M = 14; mean age 44; range 23–77 years) performed both FTT and VFMT with the hand contralateral to the lesion. We included in the study patients with evidence of glioma in peri-rolandic region and no contraindications to MRI, while we excluded patients who, for clinical reasons or poor compliance, performed FTT or VFMT exclusively. In 12 cases, tumors were localized in the right hemisphere. Seven patients presented tumor recurrence. All patients underwent surgical glioma resection by microsurgical subpial technique (17). The entire sample gave informed consent before the experiment, and the protocol was approved by the medical ethics committee of the I.R.C.C.S. Neuromed (Ethical Approval Code: 11/17 21-12-17). Clinical characteristics and histological diagnoses are shown in Table 1.

**Table 1:** Demographic and clinical data: tumor type, localization and EOR.

**Patients**			**Clinical information**		**Tumor location**			**fMRI activation localization**		**Concordance fMRI tasks—DES**				**Distance fMRI-lesion**	
										**Sensitivity**		**Sensitivity**			
	**Sex**	**Age**	**Histology (WHO grade)**	**Relapse**	**Emisphere**	**MNI AAL template**	**EOR**	**FTT**	**VMFT**	**FTT**	**VFMT**	**FTT**	**VFMT**	**FTT**	**VFMT**
1	M	45	Oligodendroglioma (II)	Yes	R	S1, SPG, precuneus	93%	M1 R, SMA R, S1 R, MFG R	M1 R, SMA R	-	-	-	-	>1cm	>1cm
2	F	38	Diffuse astrocytoma (II)	Yes	R	SFG, SMA, ACC	88%	M1 R, SMA R/L, SFG R/L, MFG R	M1 R, SMA R	-	-	-	-	<5mm	<5mm
3	M	37	Anaplastic astrocytoma (II)	Yes	R	M1, PMd, SMG	86%	S1 R, SMA L/R, M1 L, S1 L, MFG L	M1 R, SMA L	-	-	-	-	<5mm	<5mm
4	M	46	Oligodendroglioma (II)	Yes	R	M1, SMA	92%	M1 R/L, S1 R, SMA L, MFG R	M1 R	50%	100%	75%	100%	<5mm	>1cm
5	F	70	Adenocarcinoma (II)	No	L	S1	90%	S1 L, M1 L, SMA L, SMA R, M1 R, PMd R, SPG R	M1 R, SMA R	-	-	-	-	>1cm	>1cm
6	M	40	Glioblastoma (IV)	No	R	SFG, IFG, CC	92%	MFG R	M1 R	-	-	-	-	none	>1cm
7	F	55	Oligoastrocytoma anaplastico (III)	Yes	R	M1, PMd, MFG, IFG	90%	M1 R, S1 R, M1 L, S1 L	M1 R, SMA L, S1 L	-	-	-	-	>1cm	>1cm
8	M	46	Glioblastoma (IV)	No	L	SM1, SMA, SPG	76%	M1 L, S1 L, PMd L, IFG L, SMA R, M1 L, S1 L, MFG L, SFG L	M1 R	28%	100%	100%	87%	<5 mm	<5 mm
9	F	52	Glioblastoma (IV)	No	R	M1, MFG, SFG	88%	M1 R, S1 R, SMA L, SMA R, M1 L, S1 L, MFG L	M1 R, SMA R	30%	100%	100%	100%	<5 mm	<5mm
10	F	34	Anaplastic astrocytoma (III)	No	R	M1, SMA, SFG, MFG	98%	M1 R, S1 R, SMA R, SMA L, MFG L, IFG R, M1 L	M1 R, SMA L, MFG L	50%	100%	100%	100%	<5mm	<1mm
11	M	23	Ganglioglioma (I)	No	L	SMA	95%	M1 R/L, S1 R/L, SMA R/L, PMd L, SPG R, SMG R/L	M1 L, SFG L	37%	100%	100%	100%	<5mm	<1mm
12	M	51	Diffuse astrocytoma (II)	No	L	M1, S1, CC, precuneus	86%	M1 L, S1 L, M1 R, SPG R, SMG L, AG L	M1 R/L	66%	100%	100%	100%	<5mm	<1 mm
13	M	42	Glioblastoma (II)	No	R	M1	88%	M1 R/L, S1 R/L, SMA R/L, CC L	M1 R/L, SMA R	57%	100%	50%	100%	<5 mm	<5mm
14	M	33	Anaplastic astrocytoma (IV)	No	L	Thalamus, hippocampus	77%	MFG L, preSMA L	M1 L, S1 R					>1cm	>1cm
15	M	45	Glioblastoma (IV)	Yes	L	M1	84%	M1 R/L, S1 L, SMA L, SPG R	M1 R/L	71%	100%	100%	100%	<5mm	<5mm
16	M	28	Glioblastoma (V)	Yes	R	M1, SFG, MFG, IFG, insula	91%	M1 R, S1 R, IPF R, M1 L, SMA L, SFG L, MFG L	M1 R, SMA L, MFG L	-	-	-	-	<5mm	<1mm
17	M	77	Radionecrosis (III)	No	R	SM1, SPG	70%	SFG R	M1 R, M1 L, SMA L	100%	100%	50%	100%	-	<5mm
18	F	42	Radionecrosis (III)	No	L	M1	85%	S1 L, SPG R/L, SMA R/L, M1 R	M1 R/L	50%	100%	66%	100%	<5mm	<5mm
19	M	46	Glioblastoma (IV)	No	L	M1	86%	M1 R/L, S1 L, SPG R/L, SMA L, PL R	M1 R/L, S1 R	57%	100%	100%	85%	<5mm	<5mm
20	M	30	Oligodendroglioma (II)	No	R	M1, MFG, IFG	88%	M1 R/L, S1 R/L, SMA R	M1 R/L, SMA R	-	-	-	-	<5mm	<5mm

*Functional localization of fMRI maps, sensitivity, specificity and minimum distance between functional activation and lesion in both tasks. Tumor location and fMRI activation: M1, motor cortex; S1, sensory cortex; SM1, sensory-motor cortex; SMA, supplementary motor cortex; PMd, dorsal pre-motor cortex; SFG, superior frontal gyrus; MFG, middle frontal gyrus; IFG, inferior frontal gyrus; CC, cingulate cortex; SPG, superior parietal gyrus, IPG, inferior parietal gyrus; SMG, supramarginal gyrus; AG, angular gyrus*.

### Magnetic Resonance Imaging

The MRI study was performed at the Neuroradiology Unit of IRCCS NEUROMED, Pozzilli (Is), Italy. MRI data were acquired on a 3T GE Signa HDxT scanner (General Electric Medical Systems, Milwaukee, WI). A high-resolution structural T1-weighted image was acquired using a 3D spoiled gradient recalled (SPGR) sequence [repetition time/echo time/inversion time (TR/TE/TI) = 10.26/4.192/400 ms, flip angle = 15°, field of view (FOV) = 256 mm, slice thickness = 1 mm, matrix size: 256 × 256]; then, an axial fast recovery fast spin echo (FRFSE) T2 scan (TR/TE/TI = 11,002/162.92/2,250 ms, FOV = 240 mm, slice thickness = 4 mm, matrix size: 320 × 224) and 3D Fast Spin Echo T2 image (TR/TE/TI = 6,000/140.524/1,824 ms, FOV = 256 mm, slice thickness = 1.6 mm, matrix size: 320 × 320) were also acquired. Further, the same sequences were acquired post-surgery in order to evaluate the extent of resection (EOR). Blood oxygenation level-dependent (BOLD) contrast functional imaging was acquired using a whole-body radiofrequency coil for signal excitation and an eight-channel head coil for signal reception. The acquisition was performed utilizing T2^*^-weighted echo-planar imaging (EPI) sequences with the following parameters: TE 30 ms, matrix size 64 × 64, FOV 288 mm, flip angle 90°, and slice thickness 3 mm. For each subject, two different fMRI sessions were acquired, depending on the task performed by the subject. For the FTT, 100 functional volumes consisting of 39 transaxial slices parallel to the anterior commissure–posterior commissure (AC–PC) line were acquired with a TR of 3 s. For VFMT, 120 functional volumes consisting of 39 transaxial slices parallel to the AC–PC line were acquired with a TR of 2 s. All the images were anonymized. The whole acquisition period required about 35 min.

### fMRI Task Design and Paradigm

**Figure 1:**
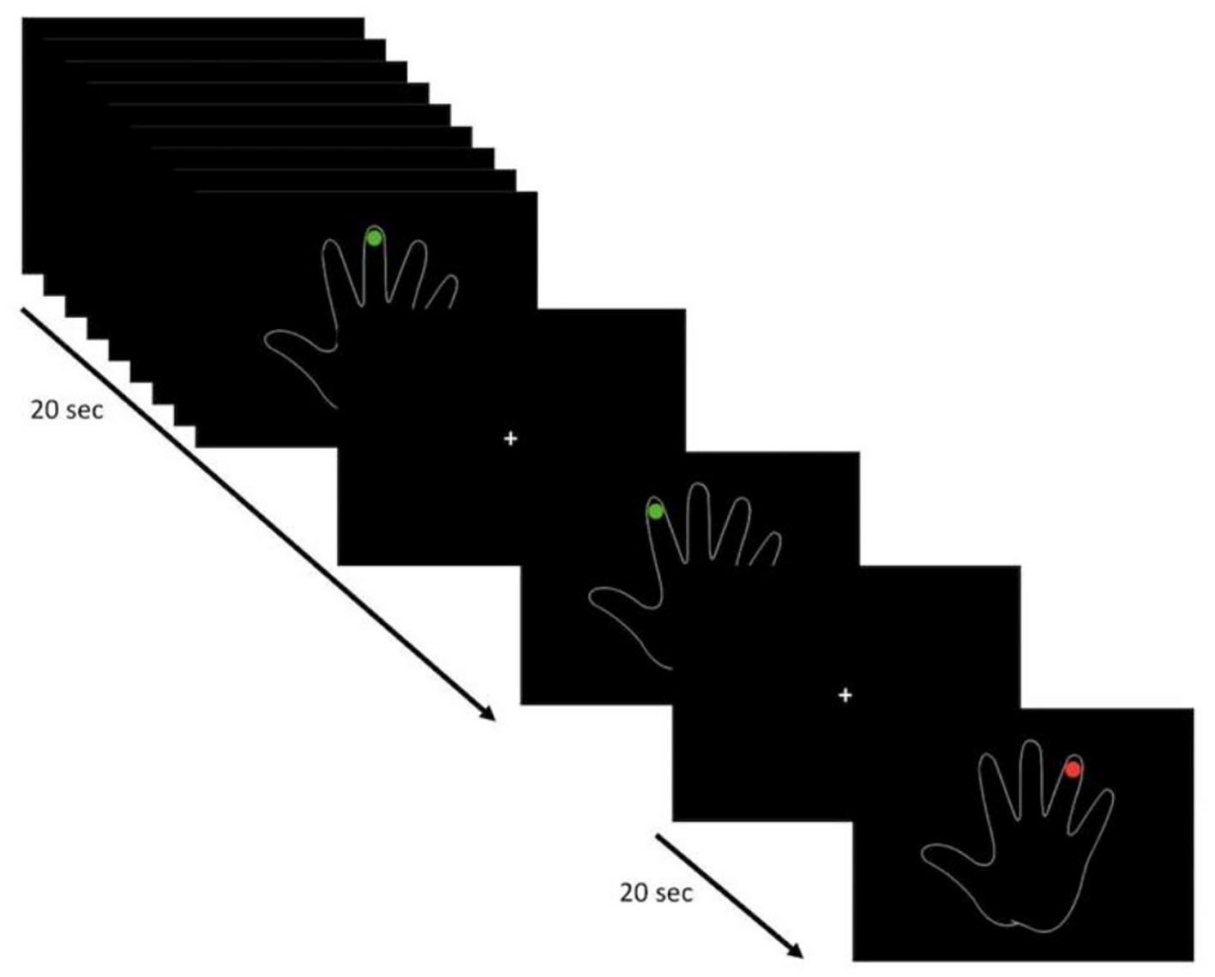
Visual-triggered finger movement task (VFMT) protocol (e.g., right hand). Active condition (green dot): patients are instructed to move the corresponding finger when a green dot appears. Passive condition (red dot): patients are instructed to observe without making any movements.

For both tasks (FTT and VFMT), fMRI acquisition was performed using a block-design paradigm. The FTT was administered over 5 min 15 s (five dummy runs) with five cycles consisting of 30 s of active condition, in which the patients executed a repetitive self-paced touch of thumb to each finger, followed by 30 s of baseline condition, in which the patients were instructed to look at a fixation point without performing any movement. The instruction of “go” or “stop” was presented via a high-quality stereo headphone set. The VFMT was administered over 4 min 10 s (five dummy runs) with six cycles consisting of 20 s of active condition, in which they observed a green dot randomly appearing on each finger, requiring the execution of abduction of the corresponding finger for a total of 10 movements (twice for each finger), followed by 20 s of baseline condition in which a red dot appeared randomly twice on each finger, with patients instructed to passively observe without executing any movements (Figure 1). VFMT was administered using E-Prime presentation software (Psychology Software Tools; www.pstnet.com) and the Nordic Neurolab visual system (Nordic NeuroLab, Bergen, Norway). A dedicated neuropsychologist instructed the patients before entering in the scanner room and monitored the execution of each task during the scanning sessions. FTT and VFMT were run in random order across patients.

### Single-Subject fMRI Data Analysis

#### Pre-surgical Planning

Real-time BOLD fMRI image processing was performed with BrainWaveRT (GE Medical Systems version 4.4). Data quality was monitored in real-time to alert the operator of poor data acquisition due to patient head movement and through task-performance monitoring with real-time activation maps. Moreover, an immediate post-processing of fMRI data was conducted on the scanner console using BrainWavePA software. An automatic pre-processing of functional scans was performed including images realignement using Woods AIR method (18, 19) to minimize movement artifact. Motion correction data indicated the magnitude and direction of rotations and translations detected and corrected during realignment. These data were extracted, and statistical comparison between the two tasks was run into R-studio software 1.3.1 performing Student's *t*-test. The fMRI image volumes were smoothed with a Gaussian spatial filter of full width at half maximum (FWHM) of 8.0 × 8.0 × 8.0 mm. A multiple regression analysis was then performed on the scans, and a *t*-test map was generated. To rate the temporal autocorrelations due to the smoothness of the hemodynamic response, the Worsley and Friston method was used, and the effective number of degrees of freedom estimated. Through the latter, the *t*-test maps were turned into an activation Z-map, and a *p*-value = 0.0001 was used for thresholding. Pre-processed functional volumes of patients were co-registered with the corresponding structural dataset, then the activation maps were created and visualized in the three orthogonal planes and in 3D rendering.

#### Intraoperative Mapping

**Figure 2:**
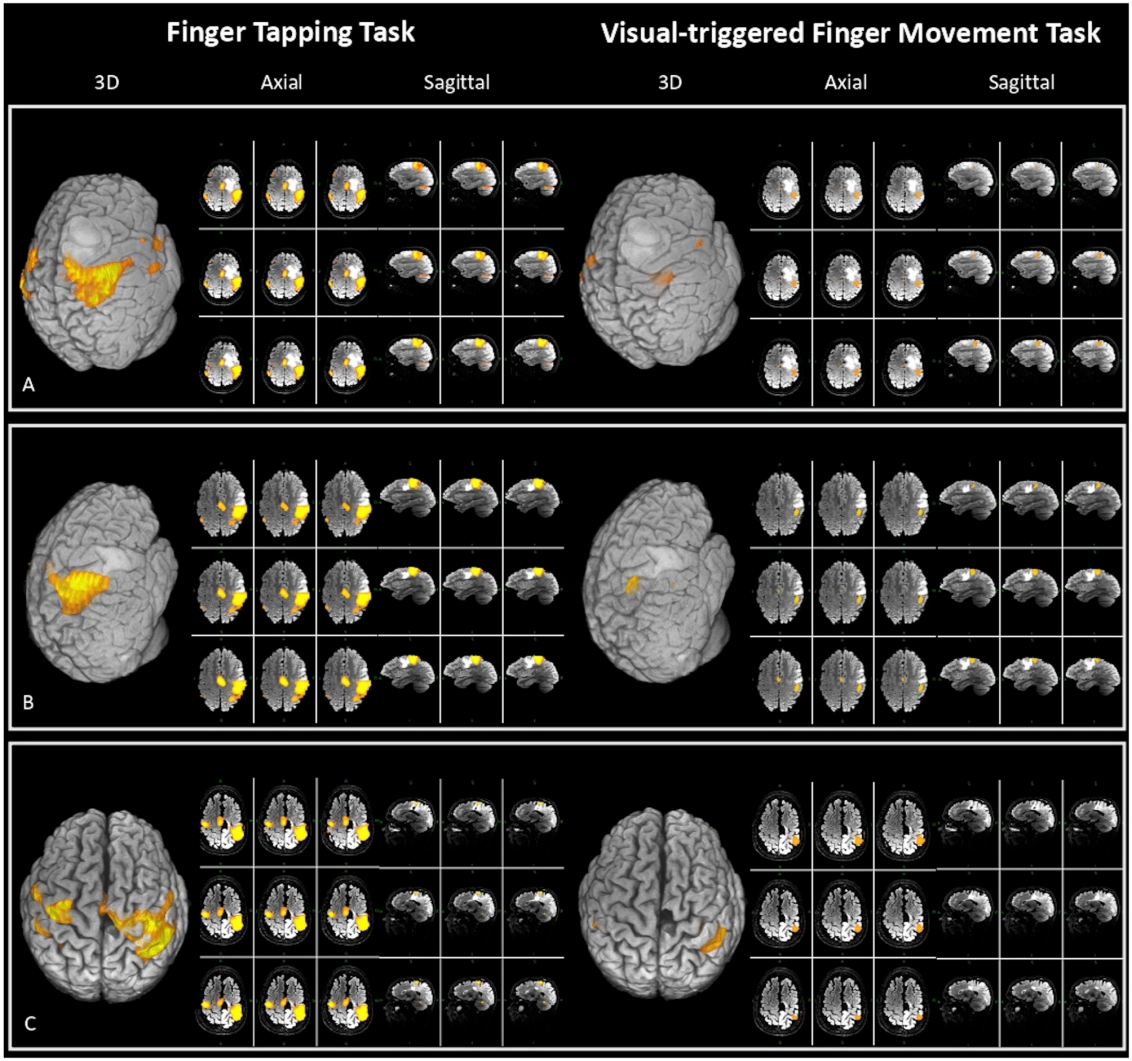
Three-dimensional (3D), axial, and sagittal views of the activation maps for both fMRI tasks [finger tapping task (FTT) on the left and visual-triggered finger movement task (VFMT) on the right], elaborated at single-subject level in three exemplar patients for clinical purpose with BrainWave software on T2 MRI sequences. The figure also displays tumor position and its spatial relation with eloquent areas. **(A)** Patient 10: closer proximity between lesion and FTT vs. VFMT activation map [fMRI specificity: VFMT 100%; FTT 50%; extent of resection (EOR) 98%]. **(B)** Patient 20: close proximity between lesion and both fMRI maps <5 mm (EOR 88%). **(C)** Patient 4: a case of tumor recurrence in which VFMT compared with FTT maps has greater reliability (fMRI specificity: VFMT 100%; FTT 50%) and has a major predictive value on EOR (92%).

In 11 patients (55% of the whole sample), craniotomy for resection allowed to expose M1. Before surgical resection, the motor cortex was stimulated through an anodal monopolar probe in order to map the cortical motor sites. DES consisted in a biphasic electrical current (60 Hz, 1 ms, 1–4 mA), which creates a “virtual transient lesion” on the cortex. When the stimulated site elicited a MEP, it was considered a positive site. We stimulated a mean of 11 sites that allowed to reach a complete cortical mapping. The DES results were compared with the 3D fMRI maps made with BrainWave for both tasks (as in Figure 2). The concordance between DES and fMRI maps was evaluated in terms of the percentage of true-positive rate (sensitivity) and true-negative rate (specificity).

#### Neuronavigation and Lesion Volume Estimation

fMRI raw data in DICOM format were imported into iPlannet server (version 3.0.1, Brainlab AG, Feldkirchen, Germany) for the neuronavigation planning. The default threshold (t-score) was set to a statistical significance of *p* = 0.001 and could be manually adapted. The proposed t-score was adjusted to reach an activation map overlapping to the one previously reconstructed in BrainWavePA software. Specifically, the threshold was increased until the cluster of activation in M1 reached the same extent obtained from BrainWave analysis.

iPlannet was also used to define tumor volume and postoperative tumor residual. These volumes were evaluated in all patients by using contrast T1-weighted or T2-weighted MR images in native space, acquired before and after tumor resection. In those cases of tumor recurrence, the previous resection cavities were estimated and not included in the lesion volume. Postoperatively, we estimated the EOR as the percentage of the resected volume compared with the pre-operative volume: EOR = (pre-operative tumor volume–postoperative tumor volume)/pre-operative tumor volume.

#### Anatomical Localization of fMRI Activation Maps

In order to find the anatomical localizations of fMRI activations, Formation of Burnt-In-Pixel (BIP) maps outlining the area of activation created with the BrainWave, together with the corresponding 3D T1 images and the 3D T2 fluid-attenuated inversion recovery (FLAIR) images were exported in DICOM format and analyzed using a custom pipeline in SPM12 environment (https://www.fil.ion.ucl.ac.uk/spm/software/spm12/). The images were first converted from DICOM to NIFTI format; then each resulting image was normalized into Montreal Neurological Institute (MNI) space using the built-in function in SPM12. After the normalization, a co-registration between the structural and functional images, including also the Automated Anatomical Labeling (AAL) template, was performed using the normalized mutual information (20). Finally, the images were smoothed using the built-in function of SPM12, with a 6-mm Gaussian smoothing kernel. In order to anatomically localize the fMRI activations, the BIP image was loaded into xjView toolbox (https://www.alivelearn.net/xjview), and the peak coordinates of each cluster were extracted (Table 1).

#### fMRI Second-Level Analysis

Differences in fMRI activations maps between the two tasks (FTT and VFMT) were investigated through a second-level fMRI analysis implemented in SPM12. To examine the difference in neural activity between the two tasks, all the contrast images for each task created from the first-level analysis in SPM12 performed on raw data were entered into a second-level two-sample t-test model. According to the hand used for task execution, we divided the sample in two groups (left hand, *n* = 8; right hand, *n* = 12), and we examined which voxels survived by selecting a cluster-forming threshold of *p* <0.001 and a cluster size of 10 voxels. Then, to localize the survived cluster of activation, they were superimposed on an AAL template in xjView.

## Results

### Pre-operative Mapping

All patients completed both fMRI tasks successfully, although two patients (patients 6 and 17) showed poor task execution performance during FTT. In three cases, the task was re-performed due to poor data acquisition. Table 1 shows the localization of single-subject fMRI activation clusters for both tasks, performed for pre-surgical planning purposes with BrainWavePA software using a conservative threshold of *p* <0.0001. In 18 patients (90%), the activation maps of VFMT were more selective than FTT maps. Specifically, FTT revealed a widespread network of activation involving areas not closely related to motor processing, whereas VFMT was able to elicit a more focal cluster of activation in M1 and other motor regions (i.e., SMA). In the two cases mentioned above, the FTT was not able to provide activation in the sensory motor cortex at a fixed threshold due to motor coordination deficit. Conversely, in patients 9 and 13, while FTT produced widespread activation maps, VFMT required a reduction of the threshold (*p* = 0.001) to obtain a cluster of activation in the hand-knob region.

### Intraoperative Mapping

Eleven patients underwent DES mapping. In the whole sample, the mean number of stimulation sites was 11 (±1.37). In VFMT, the mean sensitivity reached 100% and the specificity 97%, whereas in FTT, they were 54 and 86%, respectively (Table 1). In particular, the specificity was found to be poorer in FTT, in patients 13, 17, and 18 with tumors involving M1.

In addition, the EORs were compared with the distance from VFMT activation in M1. In nine cases (90%), the distance of the VFMT activation map from the lesion allows to achieve gross total resection (>80%). By contrast, in two cases, due to the close proximity between the lesion and eloquent hand-knob area, uncompleted resection was performed.

Taking these data together, VFMT maps showed a great predictive value in identifying functional areas than FTT (Figure 2).

### fMRI-Based Neuronavigation

fMRI raw data of both tasks were imported in Brainlab suite, and the thresholds were manually defined based on BrainWave maps. We observed the previously described difference in the extension of the activation map between the two tasks. In addition, on the threshold (t-score) of both VFMT (13.3 ± 3.62) and FTT (18.9 ± 7.11), a *t*-test and Fishers' test were performed. They revealed a significant difference (*p* <0.05) between the two fMRI tasks, with a higher threshold and greater variability in the FTT.

### fMRI Second-Level Analysis

The clusters of activation surviving the second-level two-sample *t*-test model, run on opposite comparisons between FTT and VFMT, showed wider activation maps in FTT. Notably, in the right-hand group, the activation clusters spread into prefrontal regions (mean cluster size = 11 voxels), whereas in the left-hand group, the activation spread into inferior parietal regions (mean cluster size = 28 voxels). These statistical data confirmed the more focal cluster of activation in VFMT compared with FTT.

## Discussion

Pre-operative mapping techniques are routinely used to plan surgical resection of lesions located in the cerebral central region to identify eloquent cortical areas. fMRI is a well-established and a widely available technique to obtain a pre-operative functional cortical mapping. Traditionally, the block-design FTT is implemented to map the hand-knob region, although several methodological aspects may cause a loss of specificity in localizing this area. Here, we tested a novel, clinically based VFMT able to overcome the weaknesses of FTT in the fMRI environment. We compared the accuracy of these two tasks (i) in generating the 3D maps for pre-operative planning, (ii) in the overlap of fMRI data with DES during intraoperative mapping, and (iii) in neuronavigation for fMRI image guidance.

Preliminary data of 3D single-subject functional maps seemed to show a more focal cluster of activation in the hand-knob areas for VFMT compared with FTT. Moreover, results of FTT maps showed activations in cortical regions far beyond the hand-knob area, such as the prefrontal and inferior parietal regions (Table 1). The reduced activation in the sensory cortex in VFMT, consistent with our hypothesis, could be associated with the task execution framework (i.e., no touch among the fingers), thus generating a more focal activation map with accurate localization of the primary motor cortex. By contrast, the FTT execution framework requires the touch of the thumb to each finger. This may explain the more widespread map we obtained, which included the sensory cortex and may be confounding in the localization of the hand-knob area both during the pre-surgical planning and for neuronavigation purposes.

In addition, even in intraoperative mapping data, a major accuracy of VFMT was found by comparing fMRI activation maps of both tasks to DES. Even employing a low stimulation amplitude, we observed a good concordance between the fMRI technique and DES (3, 9, 10). Sensitivity and specificity rates (100 and 97%, respectively) of VFMT were greater than those of FTT and in accordance with previous data (11, 21). By contrast, the poorer FTT sensitivity rate, compared with that in previous studies (22, 23), might be due to the lower stimulation parameters applied.

In particular, beyond the low sample size, data concerning specificity showed a greater correspondence in the activation maps of VFMT compared with FTT (Table 1). This could be seen as a better overlap between intraoperative stimulated sites and the more focal VFMT functional activation maps, whereas poor correlation with FTT maps came out. Furthermore, the more precise VFMT activation maps correlated with the EOR: indeed, in those cases in which the clusters of activation were closer to the lesion, gross total resection could not be achieved (Figures 2B,C). By contrast, the FTT activation maps' proximity to the lesion had not a predictive value on EOR (Figure 2A).

Finally, during the integration of fMRI for neuronavigation purposes, the maps generated with Brainlab software showed less extension and variability in the threshold needed to obtain a more selective map in VFMT compared with FTT. Often, operators manually reach the threshold, finding a balance between spurious and expected activation (24). Our results suggest that the operator's influence may be reduced in the manually determined threshold of VFMT. Moreover, the more accurate maps obtained from VFMT could be useful when M1 is not exposed and strip positioning for IOM is required.

To summarize, all these data may be interpreted as an overall efficacy of VFMT in the accurate localization of the hand-knob area in pre-surgical mapping and during intraoperative phases. This accuracy could be explained by some crucial novelties in the task setting. FTT indeed requires a self-paced movement of the fingers, and although the frequency of a simple motor task has not been related to fMRI signal variability (25), it is worth noting that increasing frequencies of the finger movements have been related to greater cortical activation, especially in the sensory motor cortex (26–28). Thus, on the one hand, a lower movement frequency could be associated with a poorer activation, as what emerged in two of our cases in which patients showed poor task execution performance during FTT; on the other, a greater movement rate could explain those cases with widespread activity and lower accuracy. By contrast, VFMT execution requires a randomized time-constrained finger movement. In this view, the externally paced stimuli may remove the frequency bias and may reduce the fMRI signal variability. In addition, these differences in the task execution may underlie the significantly reduced head motion degree registered during VFMT execution. It could be argued that the head motion during FTT may be increased due to the rhythmic fingers movement or even it may be reduced in VFMT due to the visual stimulus that may work as a head “anchor” requiring the patients to fixate the screen. The quality of fMRI data is strongly hampered in the presence of substantial head movements (27). Thus, it is crucial to minimize head motion to reduce artifacts and increase fMRI accuracy.

At the same time, in VFMT, the active control condition collected with the same visual stimuli of the experimental condition, but without movement execution, may allow to better isolate the primary motor cortex responses. By contrast, FTT offers a lower control due to a rest condition of “no task” (29). Moreover, beyond the voluntary movement activation *per se*, VFMT randomized cues require a continuous refresh of the movement plan, in contrast to the pacing task in FTT. Interestingly, the more widespread activation of FTT maps compared with the VFMT maps at a single-subject level emerged in our preliminary results and was confirmed by the second-level analysis run at the group level. Indeed, functional maps in FTT identified further areas in the prefrontal and inferior parietal regions within the right- and left-hand groups, respectively.

Either clinical results or ease of application could make VFMT a powerful tool in hand-knob localization for pre-surgical planning. The improvement of spatial relation knowledge about the tumor and eloquent areas may have a predictive value on EOR and be useful for ensuring safer surgery. Data acquisition is less time-consuming than other pre-operative mapping techniques, resulting in even less stress for the patients. Image processing in the clinical fMRI software is easy to perform and could be quickly implemented into clinical brain mapping routine of several neurological deficits, such as stroke, epilepsy, and Parkinson disease. Moreover, the clinical BrainWave software allows real-time monitoring of fMRI data quality, preventing poor data acquisition (30). Finally, co-registration between functional volumes and the corresponding structural dataset provides 3D maps with an accurate surface reconstruction to directly compare it with the anatomical landmarks on the exposed brain cortex intraoperatively.

## Limitations

The small sample size represents one of the limitations of the research, not allowing to evaluate the influence of tumor type and lesion size on task accuracy and reliability. Moreover, VFMT, here presented for the first time, has not been already standardized on matched control populations. The differences between fMRI task protocols could represent another limitation. Nevertheless, we adopted FTT as standardized, commonly used paradigm for hand-knob localization and designed the new VFMT in order to properly overcome its limitations, including those related to the protocol. Further, the EPI series used for fMRI acquisition may have reduced the spatial resolution. At the same time, however, it allows to replicate our study and to implement VFMT routinely in pre-surgical mapping, with clinical low-field MRI scanners (1.5T) usually employed for clinical purposes. It is worth noting that both VFMT and FTT provided no hand-knob functional activation at a fixed threshold in two patients (10%). However, in VFMT, a minimal threshold variation was sufficient to obtain a good activation map. Additionally, despite that in our sample no patients faced difficulties during task execution, severe visual impairments may result as a major limitation. We are also aware that the lack of a clinical follow-up may represent a further limitation. Nevertheless, postoperative structural MRI showed a greater VFMT predictive value on EOR, with an accurate description of the spatial relationship between lesion and tumor, in order to reach safe resection.

We designed the study to present preliminary data on the efficacy and reliability of VFMT in identifying the hand-knob area in pre-surgical mapping. Further investigations need to be conducted in the future, extending the statistical analysis to perform a standardized direct comparison between tasks on larger groups of patients and including post-surgery clinical–radiological follow-ups in order to strengthen the current scientific evidence.

## Conclusion

Pre-operative planning is a crucial step in determining surgical resection strategies of tumors involving motor cortical areas. fMRI is a widely available and well-established technique, and VFMT may represent a reliable task in localizing the hand-knob area. Thus, the more focal activation map obtained by VFMT may have a potential impact on the routine pre-surgical mapping, accurately disclosing the relationship between the lesion and the eloquent area and representing a powerful tool for surgical practice.

## Data Availability Statement

The original contributions presented in the study are included in the article/supplementary material, further inquiries can be directed to the corresponding author/s.

## Ethics Statement

The studies involving human participants were reviewed and approved by Medical ethics committee of the I.R.C.C.S. Neuromed (Ethical Approval Code: 11/17 21-12-17). The patients/participants provided their written informed consent to participate in this study.

## Author Contributions

MC, MB, and VE: design and conceptualize the study. MC, EG, LP, and GC: major role in statistical analysis. MC, EG, LP, GB, and VE: interpretation and wrote the paper. MC, EG, LP, GB, MD, PR, RM, GG, MB, SP, and VE: data acquisition. MC, EG, LP, GB, GC, PR, SP, and VE: revised manuscript. All authors contributed to the article and approved the submitted version.

## Conflict of Interest

The authors declare that the research was conducted in the absence of any commercial or financial relationships that could be construed as a potential conflict of interest.
